# Evaluation of long-term performance of an intraperitoneal biomaterial in the treatment of ventral hernias

**DOI:** 10.1007/s00464-022-09803-9

**Published:** 2022-12-22

**Authors:** John G. Linn, Eric J. Mallico, Carl R. Doerhoff, David W. Grantham, Raymond G. Washington

**Affiliations:** 1 NorthShore University Health System, 1000 Central St Suite 800, Evanston, IL 60201 USA; 2 Novant Health Bariatric Solutions—Salisbury, Salisbury, NC USA; 3 Capital Region Medical Center, Jefferson City, MO USA; 4 Pinehurst Surgical Clinic, Pinehurst, NC USA

**Keywords:** Hernia, Ventral, Hybrid, Composite, Incisional, IPOM

## Abstract

**Background:**

One-year device safety and clinical outcomes of ventral hernia repair with the GORE® SYNECOR Intraperitoneal Biomaterial, a hybrid composite mesh was evaluated.

**Methods:**

This retrospective, multicenter, case review analyzed device/procedure endpoints and patient-reported outcomes in patients treated for hernia repair ≥ 1 year from study enrollment.

**Results:**

Included were 459 patients (with 469 ventral hernias) with a mean age of 58 ± 15 years; 77.1% met Ventral Hernia Working Group 2 (VHWG2) classification. Mean hernia size was 18.9 cm^2^ and 57.3% of hernias were incisional. Laparoscopic or robotic approach was utilized in 95.4% of patients. Mesh location was intraperitoneal for 75.6% and bridging repair was performed in 57.3%. Procedure-related adverse events within 30-days occurred in 5.0% of patients and included surgical site infection (SSI), surgical site occurrence (SSO), ileus, readmission, and re-operation. Procedure-related SSI or SSO events were 3.8% through 12 months. SSO events requiring procedural intervention (SSOPI) were 2.6% through 24 months. Four patients (0.9%) had confirmed hernia recurrence through the study (the mean follow-up was 32-months, range 14–53 months). Subgroup comparisons were conducted for all type recurrence; only diabetes was found to be statistically significant (*p* = .0506).

**Conclusion:**

In this analysis, ventral hernia repair with hybrid, composite mesh results in successful outcomes in most patients. This study represents a heterogeneous patient population undergoing repair using various approaches, mesh fixation, and mesh placement locations. These data appear to confirm long-term acceptable safety and device performance with a low rate of recurrence in a predominantly VHWG2 population.

**Supplementary Information:**

The online version contains supplementary material available at 10.1007/s00464-022-09803-9.

A hernia is a defect that allows protrusion of abdominal content and can be congenital or acquired. Fifteen to 30% of patients who have a midline incision will develop a hernia [1–3]. Ventral hernia operations require a durable repair. Recurrences and complications of ventral hernia result in significant morbidity, need for additional surgery with higher failure rates, lost time and wages, decreased quality life, and poor patient satisfaction. Efforts to reduce adverse events have focused on modifying patient risk factors such as smoking, diabetes and obesity, which are established predictors of repair complications and adverse events [2–4]. Despite risk factor modifications, repair complications, including recurrence and surgical site infections, still occur [4, 5].

A durable repair requires pre-operative planning and optimization, technical expertise, mesh reinforcement, and enhanced post-operative care. Mesh implant selection remains an ongoing opportunity to affect the outcomes of recurrence, surgical site outcomes (hematoma, seroma and wound infection), as well as hernia-related quality of life [6, 7]. Implant selection is a key driver in treating hernias and has impact on the cost of treating complications when infections occur after ventral hernia repair. Intraoperative prosthetic selection may affect the outcome and whether or not explanation is necessary [8, 9]. The ability to influence both patient outcomes and the cost of ventral hernia treatment highlights the relevance of mesh investigation in hernia research [7].

Ventral/incisional hernias can be repaired using an open approach or less invasive laparoscopic or robotic approaches. Macro-porous, permanent mesh materials have been shown to have high durability, low infection rate, and low explantation rate for ventral hernia repair [9]. However, hernia recurrences and complications still occur. Absorbable mesh materials may reduce the risk of infectious complications and need for explantation, but this may be accompanied by higher long-term recurrence when compared to permanent materials [5]. Several hybrid mesh materials, constructed of both absorbable and permanent components, have been developed to address these concerns and to strike a balance between achieving high long-term durability and low long-term infection/explantation risk. Hybrid meshes can be used intraperitoneally and extra-peritoneally depending on their constructs. The GORE® SYNECOR Intraperitoneal (IP) Biomaterial (hereafter, device; W.L. Gore & Associates, Inc., Flagstaff, AZ) is a hybrid composite mesh made of a bioabsorbable 3D web scaffold and permanent dense polytetrafluoroethylene (PTFE) monofilament macro-porous knit. This device is intended for intraperitoneal placement using an underlay or intraperitoneal onlay (IPOM) technique. To secure the device, the use of non-absorbable sutures is recommended. This device is designed to result in tissue ingrowth on the 3D web scaffold surface when placed adjacent to fascia, and minimal tissue attachment on the non-porous intraperitoneal barrier film when placed adjacent to viscera [10]. Due to the lack of long-term evidence supporting these hypotheses, this study sought to analyze the long-term outcomes of this IP device for the repair of ventral and incisional hernias.

## Methods

### Study design

This multicenter, retrospective study included adult patients treated with the IP device to repair ventral, incisional, parastomal, and inguinal hernias. Data were collected from patients at least 18 years of age between April 2016 and May 2019 across four hospitals in the United States (US) who underwent hernia repair with use of the device. A record search was conducted of cases of patients treated at least 365 days prior to site initiation. Patients were categorized according to the presence of comorbidities and to the Centers for Disease Control (CDC) Surgical Wound Classification and hernia type. The analysis reported here focuses on patients with the ventral/incisional hernia type. No vulnerable populations were included in the study. The technique for hernia repair was at the medical discretion of the implanting physician. Exclusion criteria included device implantation less than 1 year prior to site initiation, use in the repair of cardiovascular defects, CDC wound class > 1, mesh placement with the anti-adhesion barrier adjacent to fascial or subcutaneous tissue, and inability to achieve sufficient mesh overlap of the hernia defect. Additional exclusion criteria included evidence of systemic infection, known wound-healing disorder, cirrhosis, current dialysis, immunosuppression, or surgical site infection (SSI) at the time of mesh placement.

Eligible patients were enrolled and within 90 days of enrollment, demographics, medical history, physical exam, adverse event and device use data were collected retrospectively from existing medical records. A patient-reported outcome (PRO) questionnaire regarding hernia recurrence was administered, and events of surgical or autopsy procedure explant of the device were captured by the site [11]. Hernia recurrence was analyzed based on clinical assessment. The protocol for this retrospective study design stated that recurrence and IP device assessment were performed per the recommendation of the treating physician. Follow-up data were measured at 1, 6, 12, 24, and 36 months postoperatively.

The study was conducted in accordance with the US Federal regulations and with Institutional Review Board approval. Given the retrospective study design, this study was not registered in a public trial registry.

### Endpoints

The three co-primary objectives were the (1) procedural—incidence through 30 days of: SSI, surgical site occurrence (SSO), ileus, readmission, reoperation, and death; (2) device—serious device incidence of mesh erosion, infection, excision/removal, exposure, migration, and shrinkage, device-related bowel obstruction and fistula, and hernia recurrence; and (3) PRO of bulge, physical symptoms, and pain. Detailed study endpoint definitions are provided in Online Resource 1.

All procedural endpoints were captured as device- or procedure-related with the exception of death, which was captured for device-related events only. Severity was captured as serious or non-serious for the SSI, SSO, and ileus events, and as serious only for readmission, reoperation, and death.

All device endpoints were captured for events that were device-related or serious in severity. The Ventral Hernia Recurrence Inventory (VHRI) is an adapted PRO patient questionnaire [12] and contains three ‘yes/no’ questions regarding symptoms that can be associated with hernia recurrence. The responses were not considered adverse events.

The secondary endpoints included SSO, SSI, bowel perforation, unexplained or chronic pain, seroma, fistula, or adhesion formation. Only device-related and events rated serious in severity were captured with the exception of the SSO and SSI which were also captured for procedure-related and non-serious events.

### Statistical method

The 95% two-sided confidence interval (CI) was calculated using the Exact Binomial Test for each estimate for the procedural, device, and PRO endpoints and included the all-enrolled patient population. Missing data were not included. A subgroup analysis of selected patient, procedure, or hernia characteristics evaluated association to all type recurrence. One parameter was the use of suture type as the use of non-absorbable sutures is recommended to secure the device.

## Results

### Patient disposition

A total of 617 patients were enrolled of which 459 had one or more ventral hernia(s) (*n* = 469), the basis for this analysis. Site enrollment distribution of patients with ventral hernia was 59.7%, 24.4%, 13.3%, and 2.6% among the four sites.

Table 1 details the demographics and the medical history and hernia characteristics at baseline. Patients were a mean age of 58 years, over half were female (53.4%), and the majority were white (82.6%) and non-Hispanic (97.4%). Per the Ventral Hernia Working Group grading system [2], 77.1% of patients were consistent with Grade 2 class (VHWG2—comorbid) and one was listed as VHWG3. All patients were of CDC Wound Class 1 (clean). Associated comorbidities included current smoker (18.7%), obesity (62.8%), diabetes mellitus (19.6%), and chronic obstructive pulmonary disease (8.1%). The mean hernia size was 18.9 cm^2^; median (IQR) hernia size was 9.0 cm^2^ (3.0, 20.0). Incisional hernias accounted for 57.3% and were located primarily in the midline (88.0%). Most patients had laparoscopic repair (84.1%) and 57.3% included bridging repairs. Intraperitoneal mesh location accounted for 75.6% of the procedures. One investigator had a preference for the trans-abdominal pre-preperitoneal (TAPP) placement (approximately 25% of devices in this study). The TAPP technique was defined as device placement just inside the peritoneum when the peritoneum was not fully intact and possible exposure to the viscera could reasonably be expected. The adhesion-resistant film side of the device was placed adjacent to peritoneal tissue and viscera.

**Table 1:** Patient demographics, baseline medical history, and baseline hernia characteristics of patients with ventral hernia

Number of patients enrolled	459
Number of Ventral Hernias	469
Demographics	*N* = 459
Female, *n* (%)	245 (53.4)
Race, *n* (%)	
White or Caucasian	379 (82.6)
Black or African American	61 (13.3)
Asian or Pacific Islander	1 (0.2)
American Indian or Alaska Native	10 (2.2)
Other	3 (0.7)
Age (years), mean (± SD)	58 (± 15)
Weight (lbs), mean (± SD)	211 (± 52)
BMI, kg/m^2^, mean (± SD) Range	33 (± 8) (15, 66)
Medical History	*N* = 459
Tobacco use, *n* (%)	
Current	86 (18.7)
Former	147 (32.0)
Never	226 (49.2)
Hypercholesterolemia	152 (33.1)
Hypertension	234 (51.0)
Diabetes mellitus	90 (19.6)
Chronic obstructive pulmonary disease	37 (8.1)
Renal insufficiency	14 (3.1)
Cancer	65 (14.2)
Cardiovascular disease	52 (11.3)
Obese	288 (62.8)
Previous surgeries and interventions, *n* (%)	*N* = 459
Renal dialysis	3 (0.7)
Abdominal aortic surgery	5 (1.1)
Ventral Hernia Characteristics	*N* = 459
VHWG Classification, *n* (%)	
Grade1: Low risk	107 (22.7)
Grade 2: Comorbid	354 (77.1)
Grade 3: Potentially contaminated	1 (0.2)
Grade 4: Infected	0
	*N* = 469
Hernia size (cm^2^), mean (± SD) Range	18.9 (± 31.7) (0.2, 276)
Hernia length (cm), mean (± SD)	4 (± 4)
Hernia width (cm), mean (± SD)	3 (± 2)
	*N* = 459
Ventral hernia, incisional only, *n* (%)	263 (57.3)
Ventral hernia, non-incisional only, *n* (%)	199 (43.4)
Device placement, *n* (%)	*N* = 459
Trans abdominal pre-preperitoneal	100 (21.8)
Intraperitoneal onlay	339 (73.9)
Repair type, *n* (%)	*N* = 459
Laparoscopic	386 (84.1)
Robotic	52 (11.3)
Open	18 (3.9)
Open conversion	3 (0.7)
Repair objective, *n* (%)	*N* = 459
Bridging	263 (57.3)
Reinforcement	197 (42.9)
Type of fixation used, *n* (%)	*N* = 459
Absorbable tacks	389 (84.8)
Absorbable sutures	188 (41.0)
Permanent sutures	171 (37.3)
Permanent tacks	43 (9.4)
Fibrin glue	4 (0.9)

*BMI* body mass index, *lbs* pounds, *SD* standard deviation, *VHWG* Ventral hernia working group

### Primary and secondary outcomes

#### 30-day procedural events, surgical site infection or occurrence, and death

Through 30 days, no deaths were reported. Procedure-related events were reported in 5.0% (23/459) of patients and reoperation in 2.4% of patients. The rate of SSI, SSO, ileus, and readmission events was 2% or less (Table 2). Through 2 years, SSOs requiring procedural intervention occurred in 2.6% (8/311; 95% CI 1.1%, 5.0%) of patients.

**Table 2:** Primary and secondary outcomes hernia

Procedure (30-day) and device (12-month) primary endpoints^a^, *n* (%)	*N* = 459
Procedure, patients with any events through 30 days, *n*/*N* (%)	23/459 (5.0) 95% CI (3.2, 7.4)
SSI	9 (2.0)
SSO	9 (2.0)
Ileus	8 (1.7)
Readmission	6 (1.3)
Reoperation	11 (2.4)
Death	0
Device, patients with any events through 12 months	0
12-Month secondary endpoints^a^, *n* (%)	*N* = 453
Patients with any events through 12 months^b^	17 (3.8)
Seroma	0
Fistula	0
SSI	10 (2.2)
SSO	14 (3.1)
Adhesion formation	0
Bowel perforation	0
Unexplained or chronic pain	0
Ventral Hernia Recurrence Inventory, *n*/*N* (%)	
Patient With “Yes” Response to: “Do you feel or see a bulge at treatment site?”	23/339 (6.8)
All known hernia recurrence	4/459 (0.9)
Recurrence: device-related SAE	1/459 (0.2)

*CI* confidence interval, *SAE* serious adverse event, *SSI* surgical site infection, *SSO* surgical site occurrence

^a^Only device-related and serious severity events were captured with exception of the SSO and SSI which were also captured for procedure-related and non-serious events

^b^Patients with multiple types of events (e.g., SSI and Ileus) would only count once for the composite endpoint in this row but would appear in multiple rows below. All rows are *not* a count of events but rather a count of patients with at least one qualifying event

#### 12-month device events

At 12 months, 452 patients were available for follow-up. No serious events of mesh erosion, infection, excision/removal, exposure, migration, or shrinkage, device-related bowel obstruction or fistula, or hernia recurrence were reported. Nor were there reports of seroma, fistula, known adhesion formation or bowel obstruction, bowel perforation, or unexplained or chronic pain (Table 2). The percentage of patients with SSI and SSO were 2.2 and 3.1%, respectively.

### Safety and patient-reported outcomes

Through 3 years, 18.1% (83/459) of patients had 137 adverse events. In 13.7% (63/459) of patients, 101 procedure-related adverse events were reported. Four patients (0.9%) had treatment for an adverse event recurrence that involved the device or the originally treated hernia.

#### Serious adverse events and deaths

After 3 years, 72 serious adverse events (SAE) were reported in 52 patients (11.3%). Of patients experiencing an SAE, 67.3% were classified as procedural complications; namely, procedural pain (29 patients/29 events) and postoperative ileus (6 patients/6 events). A total of 11 deaths were reported at the 6-months (*n* = 2), 1-year (*n* = 4), 2-year (*n* = 2) and 3-year (*n* = 2) follow-up periods and 1 death with an unknown time period; none were procedure- or device-related. Of note, one procedure-related SAE involved a patient with a device explant on day 16 post-implant in which imaging observed free air suggestive of preperitoneal abscess with infected mesh. The explanted tissue and mesh were cultured by the hospital and study sponsor, each returned negative results for infection, and therefore, was listed as a procedure-related adverse event.

#### Recurrence

Sites reported 4/459 (0.9%) clinical recurrences. One was a non-serious procedure-related event. Three were classified as an SAE: one each was device-related, procedure-related, or not at the same treatment location of the device. Only 1 (0.2%) of the patients met the protocol description for recurrence for the device endpoint. This recurrence was a 4 × 4 cm recurrent hernia that occurred lateral to the prior repair of a periumbilical hernia. Original mesh placement was IP and used a bridging technique with a single piece of mesh and absorbable sutures and tacks. The patient recovered on Day 429 post-implant.

The VHRI PRO questionnaire was completed by patients > 12-months post-procedure. Data were captured at 2 years (*n* = 93), 3 years (*n* = 113), and beyond (*n* = 133). Through 53 months, a ‘*yes*’ response for questions of “bulge at the treatment site” was reported by 6.8% of patients. The median follow-up time was 33 months (range 14–53 months).

#### Subgroup analyses

Multiple subgroup analyses were performed to evaluate any potential association to all type recurrence. Only patients with diabetes 9/70 had a numerically higher risk for all type recurrence than non-diabetes 17/282 (*p* = 0.0506), which approached but did not reach statistical significance. Subgroup comparisons for all other parameters tested were not statistically significant (Table 3).

**Table 3:** Subgroup comparison for all type recurrence

Parameter	Yes, *n*/*N* (%)	No, *n*/*N* (%)	*p*-value
Diabetes	9/70 (12.9)	17/282 (6.0)	0.05
Obesity	17/213 (8.0)	7/118 (5.9)	0.49
Smoking (history)	16/176 (9.1)	10/176 (5.7)	0.22
Incisional	14/189 (7.4)	11/150 (7.3)	0.98
Laparoscopic repair	24/315 (7.6)	1/24 (4.2)	0.53
Midline involvement	25/339 (7.4)	1/13 (7.7)	0.97

Parameter	*n*/*N* (%)	*n*/*N* (%)	*p*-value
VHWG	VHWG 2 21/257 (8.2)	VHWG 1 4/81 (4.9)	0.33
Hernia	< 9 cm^2^ 12/180 (6.7)	≥ 9 cm^2^ 14/172 (8.1)	0.60
Device placement	Preperitoneal 3/81 (3.7)	Intraperitoneal 22/258 (8.5)	0.15
Fixation	Permanent 11/152 (7.2)	Absorbable 14/187 (7.5)	0.93
Repair objective	Bridging 14/190 (7.4)	Reinforcement 11/149 (7.4)	1.0
IPOM	With bridging 12/132 (9.1)	With reinforcement (IPOM plus) 10/120 (8.3)	0.83

*IPOM* intraperitoneal onlay, *VHWG* Ventral Hernia Working Group

## Discussion

This study reviewed 459 patients with 469 ventral hernias treated with the IP device for long-term (mean follow-up of 32-months) evidence that might support the use of the IP hybrid biomaterial for ventral and incisional hernia repair. In this population, 77% of patients were VHWG2. It is well-established that mesh implant selection factors affect outcomes of hernia repair, particularly for recurrence and post-operative complications as well as cost and quality of life [7–10].

The early rate of procedure-related adverse events of SSI, SSO, ileus, readmission, and re-operation with the IP device were low, 5% in this study. Long-term, 3% or less of patients had SSI or SSO events. Through 24 months, 2.6% of SSO events required SSOPI. However, while the mesh types were not reported, the Baucom et al. [11] paper evaluated 632 patients and had an SSOPI rate of 9.8% through 2-years. It is important to note, that SSI was not found to be the reason for mesh removal in this study. For our study, this may suggest that the overall treatability of the IP device may mitigate the need for its removal if a postoperative infection were to occur.

There were no reports of hernia recurrence in the first year. However, through 53 months, 6.8% of patients self-reported recurrence though only one patient had a clinically confirmed device-related recurrence classified as an SAE. With or without mesh and regardless of repair site, recurrence is a known risk [5, 7, 8, 13, 14]. Although the appropriate size of mesh overlap and mesh-to-defect (M/D) ratio in hernia repairs is debatable, the median (IQR) M/D ratio of 20 (13 to 43) in this study is above the “safe minimum” M/D ratio of greater than 16 reported by Hauters et al. [15], based on a prospective series of laparoscopic ventral hernia repairs using a bridging technique. Bridging repairs are associated with a higher risk of recurrence [16]. In this study, 57% were bridging repairs. The collection of the data retrospectively did not allow for insight as to surgeon preference. However, even with presumed greater risk of recurrence, the overall rate of clinical recurrence in our study was < 1%. Ventral hernia repair comes with an inherent risk and multiple factors may be associated with decreased success for these types of procedures. Other studies have demonstrated that some hybrid mesh materials performed less favorably than the mesh material in this study. One such study looked at long-term performance of the TIGR® matrix, a resorbable polylactide, polyglycolide, and trimethylene carbonate composite mesh. Of the 91 patients evaluated, the rate for recurrence was 12% and for wound complications was 27%; the mean follow-up was 42.4 months (range 0–102 months) [13]. A prospective, multicenter, single-arm post-market trial study evaluated Zenapro™, a composite mesh of polypropylene and small intestine submucosa, [14] in 63 patients, 87.3% of which were VHWG2. At 12 months, rates were 6.8% for recurrence, 3.4% for SSI with a procedural intervention, and 23.7% for seroma. Our study observed favorable outcomes of low rates of recurrence, AE, infection, SSOPI, seroma and explant compared to these clinical studies.

The IP device used in this study is a hybrid made of a bioabsorbable 3D web scaffold and permanent dense PTFE monofilament macroporous knit. The low seroma rates in this study suggest that use of this material may promote tissue ingrowth, which leads to low infection and low hernia recurrence and was observed in this study as only one device-related SAE recurrence was observed in clinic. The IP device appears to have less microbial colonization than reported with Zenapro®, Ovitex® 1S Permanent and Ovitex® 1S Resorbable [17].

Patient risk factors such as smoking, diabetes, and obesity have been established as predictors of repair failure and decreased success [4]. Our study patient population included risk factors of smoking, diabetes, and obesity for which only diabetes was found to be a significant predictor for all type recurrences. Smoking prior to hernia surgery is known to be an indicator of poor outcomes [4]. In this study, smoking cessation was based on investigator discretion and represents what likely happens in the general US population.

Applicability of this data to patient populations in Europe is feasible with an understanding of regional differences. As a US-based study, the patient population tended to be more obese compared to an appreciated European Union population. This has the potential to overestimate the rate of recurrence and wound complications compared to the anticipated experience within Europe [17, 18].

## Limitations

Our study has limitations, namely the retrospective data collection. The data are dependent on standard documentation for procedures as well as thorough chart review. The evaluations of hernia recurrence and performance of the IP device were not standardized and were based on clinical assessment. Ideally, a radiological exam is preferred. The retrospective design called for assessment per the recommendation of the treating physician. Retrospective data collection is well represented in hernia mesh literature that attempts to describe long-term outcomes, particularly with recurrence and mesh explantation. The long-term outcome data collected in this study were supported by the performance of a remote survey specific to hernia pathology and reflective of a patient’s perspective on their care. The results are further limited from the lack of comparison data of the IP device to other types of mesh products. The next limitation is the choice of quality of life and patient-reported outcome tool. The use of Patient Reported Outcome Metrics (PROM) tool has potential to capture longer term mesh experience data as well as to adjust hernia surveillance schedules according to perceived risk. In this study, inclusion of a PROM was suggestive for further investigation to confirm recurrence.

Our study did not identify any new, device-specific risks or complication rates that would be deemed as unacceptable. The totality of collected data regarding the real-world performance of this IP device supports that it is a viable treatment option for physicians who prefer a permanent synthetic mesh as part of laparoscopic, robotic or open repair of ventral/incisional hernia.

## Conclusion

In this analysis, ventral hernia repair with a hybrid composite IP mesh results in successful outcomes in the majority of patients. This study represents a heterogeneous patient population undergoing repair using various surgical approaches, mesh fixation, and mesh placement locations. These data appear to confirm long-term acceptable safety and device performance with a low rate of recurrence in a predominantly VHWG2 (comorbid) population.

## Supplementary Information

Below is the link to the electronic supplementary material.

Supplementary file1 (DOCX 20 KB)
