# Correction: Evaluation of long-term performance of an intraperitoneal biomaterial in the treatment of ventral hernias

**DOI:** 10.1007/s00464-023-10057-2

**Published:** 2023-04-05

**Authors:** John G. Linn, Eric J. Mallico, Carl R. Doerhoff, David W. Grantham, Raymond G. Washington

**Affiliations:** 1 NorthShore University Health System, 1000 Central St Suite 800, Evanston, IL 60201 USA; 2 Novant Health Bariatric Solutions—Salisbury, Salisbury, NC USA; 3 Capital Region Medical Center, Jefferson City, MO USA; 4 Pinehurst Surgical Clinic, Pinehurst, NC USA

**Correction to: Surgical Endoscopy** 10.1007/s00464-022-09803-9

The original online version of this article was revised to correct an error in the Discussion section, related to the presentation of data regarding the mesh-to-defect (M/D) ratio in hernia repairs. The correct M/D ratio is 20 (13 to 43).

The original article has been corrected.

