# Correction: Evaluation of long-term performance of an intraperitoneal biomaterial in the treatment of ventral hernias

**DOI:** 10.1007/s00464-023-10079-w

**Published:** 2023-04-21

**Authors:** John G. Linn, Eric J. Mallico, Carl R. Doerhoff, David W. Grantham, Raymond G. Washington

**Affiliations:** 1 NorthShore University Health System, 1000 Central St Suite 800, Evanston, IL 60201 USA; 2 Novant Health Bariatric Solutions—Salisbury, Salisbury, NC USA; 3 Capital Region Medical Center, Jefferson City, MO USA; 4 Pinehurst Surgical Clinic, Pinehurst, NC USA

**Correction: Surgical Endoscopy** 10.1007/s00464-022-09803-9

The original online version of this article was revised to correct the units of data in the Results section and Table 1. Data in the Results section was originally presented in cm and should have been presented in cm^2^. Data in Table 1 was originally presented in cm^3^ and should have been presented in cm.

The original article has been corrected.

