# Supplementary material for: Evaluation of long-term performance of an intraperitoneal biomaterial in the treatment of ventral hernias
Source: Surg Endosc. 2022 Dec 22;37(5):3455–62. doi: 10.1007/s00464-022-09803-9 (PMC10156806; doi:10.1007/s00464-022-09803-9)
Supplement: Supplementary file 1 — Supplementary file1 (DOCX 20 KB) [file 464_2022_9803_MOESM1_ESM.docx]

**Online Resource 1. Study Endpoint Definitions**

| **Term** | **Study Endpoint Definition** |
| --- | --- |
| Death | Death that may be associated with either a procedure or device-related adverse event within 1 month after the operation |
| Hernia Recurrence | Loss of surgically acceptable hernia repair at the original treatment site |
| Hernia Recurrence requiring intervention | Repeat operation that may have been due to loss of surgically acceptable hernia repair at the original treatment site |
| Hernia mesh-related reinterventions | A second operation in the area of the hernia repaired initially to address complications that may have been caused by or associated with the mesh |
| Readmission | An unplanned hospitalization that may have been related to either the device or procedure with a length more than 24 hours in length |
| Reoperation | A device or procedure-related surgical or interventional procedure |
| Surgical Site Infection (SSI) CDC Definitions^a^ | Superficial Incisional SSI   - Infection occurs within 30 days after the operation; - Infection involves only the skin or subcutaneous tissue; and - At least 1 of the following:   - Purulent drainage (culture documentation not required);   - Organisms isolated from fluid/tissue of superficial incision;   - At least 1 sign of inflammation (e.g. pain or tenderness, induration, erythema, local warmth of the wound);   - Wound is deliberately opened by the surgeon; or   - Surgeon or attending physician declares the wound infected - Do not report the following conditions as SSI^b^:   - Stitch abscess (minimal inflammation and discharge confined to the points of suture penetration)   - Infection of an episiotomy or newborn circumcision site.   - Infected burn wound   - Incisional SSI that extends into the fascial and muscle layers (see deep incisional SSI)   **Deep Incisional SSI**   - Infection occurs within 30 days of operation or within 1 year if an implant is present; - Infection involves deep soft tissues (e.g. fascia and / or muscle) of the incision; and - At least 1 of the following:   - Purulent drainage from the deep incision but without organ/space involvement;   - Fascial dehiscence or fascia is deliberately separated by the surgeon due to signs of inflammation;   - Deep abscess is identified by direct examination, during reoperation, by histopathology, or radiologic examination; or   - Surgeon or attending declares deep incisional infection is present   **Organ/Space SSI**   - Infection occurs within 30 days of operation or within 1 year if an implant is present; - Infection involves anatomic structures not opened or manipulated by the operation; and - At least 1 of the following:   - Purulent drainage from a drain placed by a stab wound into the organ / space;   - Organisms isolated from organ/space by aseptic culturing technique;   - Identification of abscess in the organ/space by direct examination, during reoperation, by histopathological examination; or   - Diagnosis of organ/space SSI by surgeon or attending physician |
| Surgical Site Occurrence (SSO)^c^ | Complications at the surgical site e.g., seroma, wound dehiscence or formation of enterocutaneous fistula |

Abbreviations: CDC=Centers for Disease Control; SSI=surgical site infection; SSO=surgical site occurrence ^a^ Report infection that involves both superficial and deep incision sites as deep incisional SSI; Report an organ/space SSI that drains through the incision as a deep incisional SSI. Horan TC, et al*. Am J Infect Control.* 1992t;20(5):271-274.

^b^ Mangram AJ, et al. *Infect Control Hosp Epidemiol*. 1999;20(4):250-78.

^c^ Breuing K, et al. *Surgery*. 2010;148(3):544-58.
